# Renal Mucormycosis: Post-COVID-19 Infection Presenting as Unilateral Hydronephrosis in a Young Immunocompetent Male

**DOI:** 10.1155/2022/3488031

**Published:** 2022-07-21

**Authors:** Rabin Nepali, Shreya Shrivastav, Dibya Singh Shah

**Affiliations:** ^1^Department of Nephrology and Transplant Medicine, Tribhuvan University Teaching Hospital, Kathmandu, Nepal; ^2^Department of Pathology, Tribhuvan University Teaching Hospital, Kathmandu, Nepal

## Abstract

Mucormycosis is a rare invasive fungal infection with a high mortality rate caused by members of the family Mucoraceae. It mainly affects immunocompromised hosts such as poorly controlled diabetes mellitus, previous solid organ transplant, high-dose steroids, and hematologic malignancy. The most common sites of the disease are rhinocerebral, the skin, the lungs, and the gastrointestinal tract. In this era of COVID-19 infection, there has been a significant rise in invasive mucormycosis predominantly reported from southeast. We present a case of isolated renal mucormycosis in an apparently healthy individual with post-COVID-19 infection presenting as unilateral hydronephrosis. Timely identifying at-risk populations and having a high degree of suspicion with involvement of multidisciplinary teams are of utmost importance to diagnose and treat a rare and fatal infection. Even if there is a long history, antifungal drugs and removal of the source can result in a good outcome.

## 1. Background

Mucormycosis is an invasive fungal infection caused by members of the family Mucoraceae [1]. The disease is known to affect patients with immunocompromised status from a variety of causes such as diabetes mellitus, hematologic malignancy, and HIV infection. The most common form of presentation is rhinocerebral infection although primary infections of the skin, the lungs, and the gastrointestinal tract have been reported [2]; however, isolated presentation of renal mucormycosis is rare [3].

With the ongoing COVID-19 pandemic and the use of high-dose steroids and antibiotics, there have been increasing reports of bacterial and fungal coinfections in the COVID-19 positive group of patients [4]. We report a rare case of isolated renal mucormycosis, post-COVID-19 infection, in an apparently healthy individual presenting as unilateral hydronephrosis.

## 2. Case Presentation

A previously healthy 39 years old male presented to us with complaints of burning micturition and a weak stream of urine for 1 month. He did not give a history of diabetes mellitus, hypertension, or other chronic illness. However, 5 months ago, he gave a history of being diagnosed with COVID-19 infection after developing fever and shortness of breath for 3 days. He was admitted at first to a private hospital for shortness of breath where he received IV dexamethasone along with other supportive treatment during which he developed high blood sugar and was shifted to the intensive care unit of another private hospital. During the treatment in the intensive care unit, he developed left-sided hemopneumothorax with bronchopleural fistula which was managed with chest tube insertion with negative suction. His sputum culture showed *Citrobacter koseri* and *Coagulase-negative staphylococcus* along with *Pseudomonas aeruginosa* in his blood culture for which he received multiple broad-spectrum antibiotics. After 6 weeks of treatment, he started having burning micturition and a weak urinary stream during micturition. He was again referred to another centre for further treatment where he underwent abdominal ultrasound which showed a polypoidal urinary bladder mass involving the right vesicoureteric junction and right hydroureteronephrosis. For this finding, he was referred to our centre. On evaluation, his hemoglobin was 10.6 gm/dl, total count of 7500/cmm, and platelets of 363000/cmm. His renal function test and liver function test were within normal limits with random blood sugar of 12 mmol/L. His urine routine and microscopy examination showed plenty of pus cells per high-power field, but his urine culture was sterile.

**Figure 1:**
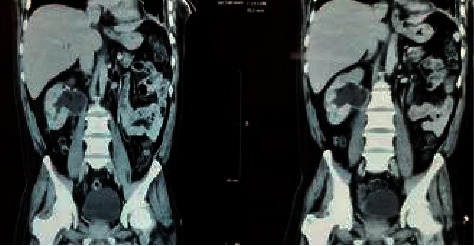
CT abdomen showed right-sided hydronephrosis.

**Figure 2:**
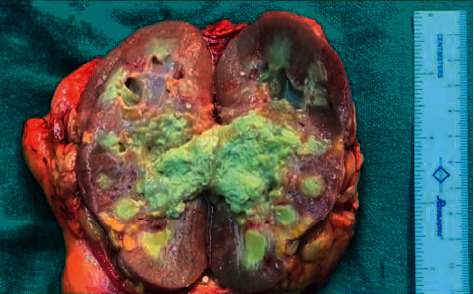
The cut section of the right kidney shows cheesy material infiltrating the renal pelvis and calyces.

**Figure 3:**
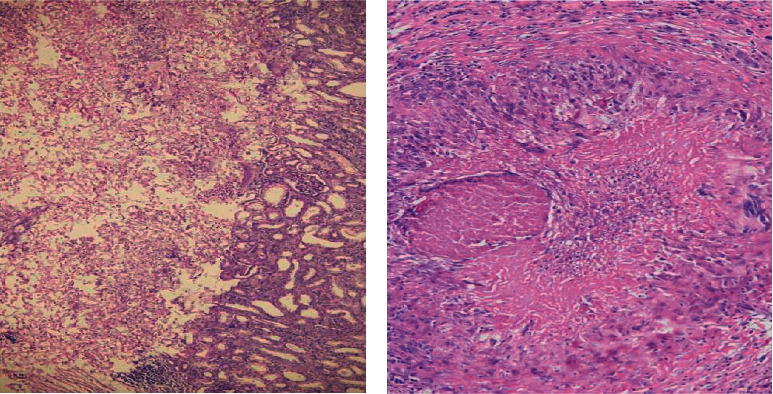
(a) Renal parenchyma with abrupt necrosis and presence of broad-based, aseptate fungal hyphae showing 90° branching (HE × 100). (b) Invasion of the blood vessel by similar fungal profiles (PAS × 200).

His contrast CT abdomen showed thickening and enhancement of the right distal ureter and the adjacent vesicoureteric junction (VUJ) region along with right distal hydroureter, and the report suggested features of a malignant lesion in the right VUJ and the distal ureter. It also showed right moderate hydronephrosis with pelvic ureteric junction narrowing and multiple nonenhancing hypodensities in the inter and lower pole cortex of the right kidney with perinephric fat strandings suggestive of chronic pyelonephritis (Figure 1). Suspecting malignancy, cystoscopy was carried out which showed necrotic mass peeping from the right ureteric orifice. The histopathological examination of the sample showed fungus with broad-based, aseptate hyphae suggestive of mucormycosis. He was then started on amphotericin B deoxycholate (1 mg/kg/day). His serum creatinine rose to a maximum of 330 micromol/L during his stay. Amphotericin B deoxycholate was changed to liposomal amphotericin B. His serum creatinine subsequently decreased to 160 micromol/L before his surgery. He underwent right radical nephroureterectomy (Figure 2). The histopathological examination confirmed mucormycosis (Figures 3(a) and 3(b)). He was continued on oral posaconazole. His serum creatinine at discharge was 113 micromol/L. In follow-up of 1 month in OPD, he was doing fine with stable renal function.

## 3. Discussion

Mucormycosis is a rare infection characterized by invasion of vasculature resulting in infarction and necrosis of affected tissues [5]. Infection is almost exclusively found in immunocompromised hosts with the most common conditions being poorly controlled diabetes mellitus, previous solid organ or bone marrow transplant, high-dose steroids, and hematologic malignancy [6].

Renal mucormycosis in apparently healthy individuals is a very rare occurrence [7], and it typically develops from hematogenous spread in disseminated cases [8]. Isolated renal involvement is even more uncommon and speculated to be caused by ascending infection of the urinary tract [9]. Isolated renal mucormycosis usually presents with fever (88%), flank pain (70%), and hematuria or pyuria (70%) [6] with concomitant bacterial urosepsis in 53% [10]. Our case of renal mucormycosis had a rare presentation of unilateral hydronephrosis and pyelonephritis. Without early diagnosis and immediate aggressive treatment, renal mucormycosis is almost universally fatal [11].

In this era of COVID-19 infection, there has been a significant rise in invasive mucormycosis predominantly reported from Southeast Asia, especially India [12]. COVID-19 infection causes innate immune dysregulation which along with increased use of corticosteroids [13] and other immunosuppressants can lead to secondary bacterial and fungal infections. Along with these factors, the patient being in mechanical ventilation, administration of industrial-grade oxygen, use of multiple broad-spectrum antibiotics, prolonged hospital stay, exposure to invasive procedures, and generally poor hygiene of the patients also provide the perfect setting for secondary fungal infections [14]. Hyperglycemia also seems to be one of the predisposing factors which along with low pH leads to dysfunction of the polymorphonuclear cells and defective intracellular killing by both oxidative and nonoxidative mechanisms [15].

Definitive diagnosis is performed by positive cultures and histopathological demonstration of thick, nonseptate hyphae branching at the right angle [16]. Treatment of renal mucormycosis includes antifungals; amphotericin B, posaconazole, and isavuconazole are the only systemic antifungal drugs with activity against Mucorales, surgical debridement of the infected tissue (nephrectomy), and treatment of any predisposing condition [17, 18].

Given the high morbidity and mortality of these patients, a high degree of suspicion in appropriate clinical settings is of utmost importance in these cases for early diagnosis and treatment even if it is in an unusual location like in our case. The number of hospitals, multiple broad-spectrum antibiotics, high blood sugar levels, steroid administration, post-COVID-19 status, and lack of expertise in previous hospitals all played a significant role in delayed diagnosis of mucormycosis that could have been fatal. Computed tomography of the abdomen, cystoscopy, and histopathological examination should be performed urgently to reach a proper diagnosis. Forming multidisciplinary teams including nephrologists, urologists, pathologists, infectivologists, and microbiologists is essential for prompt management. Despite the high fatality in these cases, if proper treatment with antifungal agents and removal of the source by appropriate operation can be performed, a good outcome can be assured.

## 4. Conclusion

Timely identifying at-risk populations and having a high degree of suspicion with involvement of multidisciplinary teams are of utmost importance to diagnose and treat a rare and fatal infection like isolated renal mucormycosis presenting as unilateral hydronephrosis. The use of antifungal drugs and removal of the source can result in a good outcome.

## Data Availability

The data supporting the findings of the study are available within the article and can be obtained from the corresponding author upon request.

## Abbreviations

HIV: Human immunodeficiency virus
Urine RE/ME: Urine routine and microscopic examination
CT: Computed tomography
VUJ: Vesicoureteral junction
PUJ: Pelvic ureteric junction.
